# Efficient Self-Condensation of Cyclohexanone into Biojet Fuel Precursors over Sulfonic Acid-Modified Silicas: Insights on the Effect of Pore Size and Structure

**DOI:** 10.1021/acssuschemeng.4c01956

**Published:** 2024-06-24

**Authors:** Antonio Martín, Esther Arribas-Yuste, Marta Paniagua, Gabriel Morales, Juan A. Melero

**Affiliations:** † Chemical and Environmental Engineering Group. ESCET, Universidad Rey Juan Carlos. c/Tulipán s/n, 28933 Móstoles, Spain; ‡ Instituto de Tecnologías para la Sostenibilidad (ITPS). ESCET, Universidad Rey Juan Carlos. c/Tulipán s/n, 28933 Móstoles, Spain

**Keywords:** mesoporous silica, sulfonic acid catalysts, aldol condensation, cyclohexanone, biojet fuel

## Abstract

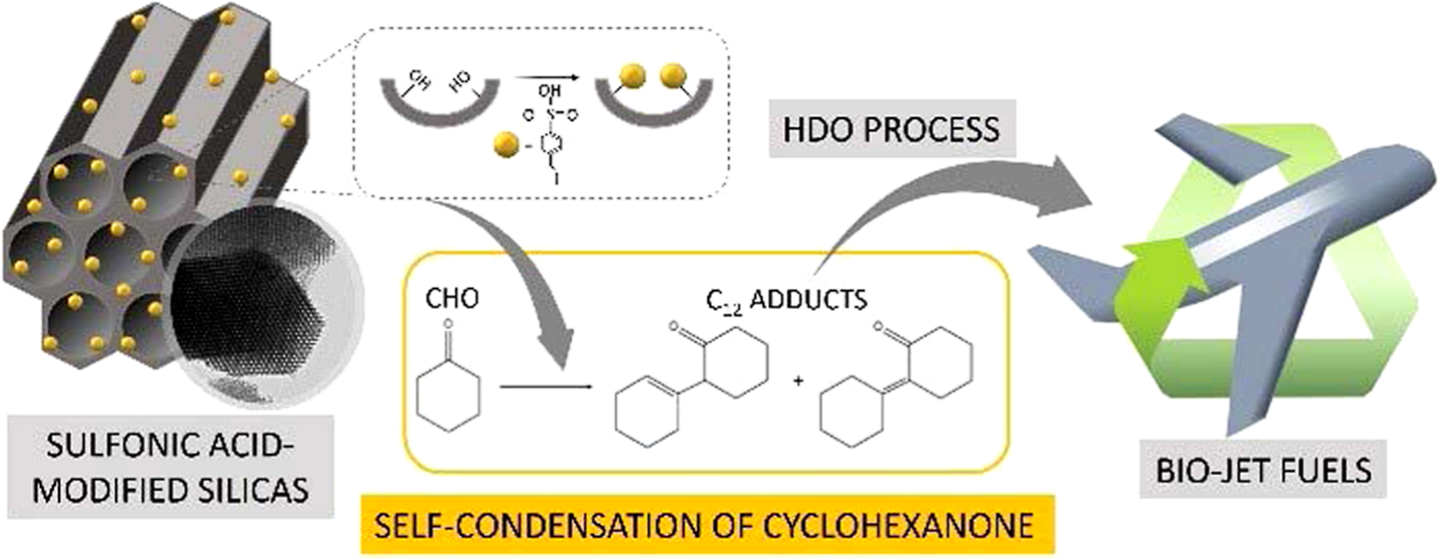

Mesoporous silica materials with different pore structures and sizes have been used for supporting aryl sulfonic acid catalytic sites via a postsynthetic grafting approach. The synthesized materials have been evaluated in the solventless acid-catalyzed self-condensation of cyclohexanone (CHO) to obtain the corresponding C_12_ adducts. These compounds display great potential as oxygenated fuel precursors as they can be transformed into jet fuel range alkanes in a subsequent hydrodeoxygenation process. In this work, the synthesized catalysts have displayed high selectivity values toward monocondensed compounds (>95%), thus limiting the formation of undesired heavier condensation products, together with CHO conversion values in the range 20–40% after 2 h of reaction at 100 °C. The structural and textural properties of the supports play an important role in the catalytic performance. Moreover, the activity per acid center is correlated with the textural properties of the supports, indicating that a lower surface density of the anchored aryl sulfonic groups affords an improvement in their specific activity. Finally, the benefit of using supports with large pore sizes and open structures, which limit the fouling of the catalysts by organic deposits, is demonstrated in a stability and reusability test.

## Abstract 2

Sulfonic acid-modified silica materials with different pore sizes and structures as heterogeneous acid catalysts in self-condensation of cyclohexanone to obtain oxygenated adducts with potential as precursors of biojet fuel range hydrocarbons.

## 1 Introduction

In the last decades, the energy demand has been continuously rising due to the sustained growth of world population and global economy, being so far mainly met by fossil fuels.^1^ The transportation sector plays a key role in the increase of energy demand as it nowadays accounts for nearly one-quarter of the global energy-related CO_2_ emissions.^2^ Particularly, one of the greatest challenges currently in this field is the development of a sustainable alternative for powering the aviation sector, where the use of electric or hydrogen-based technologies is still far from commercial application. The aviation sector utilizes approximately 3% of the world’s fossil fuels, generates around 2% of greenhouse gas emissions, and accounts for around 11–12% of all transportation-related CO_2_ emissions.^3,4^ In the current context, with a constant increase in fossil fuel prices, together with increasing environmental concerns and regulations all over the world, the development of sustainable biofuels is one of the best alternatives for replacing traditional jet fuel in the aviation sector. Thus, the European Green Deal sets out the need to reduce air transport emissions by 50% by 2050 as compared to 2005,^5^ specifically including the promotion of sustainable aviation fuels (SAFs), fully compatible with existing infrastructures and engines, as one of the key solutions.^6^ More recently, EU Refuel Aviation regulation (approved in 2023, Oct) obliged the gradual increase of the blend of SAFs from 2% in 2025 up to 70% in 2050.

However, the availability of sustainable biomass is currently a limiting factor for the large-scale development of biomass-derived SAFs. Current technology allows for the production of biojet fuels from different raw materials such as oleaginous and lignocellulosic biomass.^7^ Unlike oleaginous feedstock, lignocellulosic waste offers excellent advantages for the large-scale production of biojet fuels due to the low cost and high worldwide availability. Lignocellulosic biomass can be converted to liquid fuels by different routes: hydrothermal, catalytic, gasification, and pyrolysis. From biomass pyrolysis, furans, ketones, aldehydes, and lignin-derived phenols can be obtained.^8^ In particular, cyclohexanone (CHO) is a selective hydrogenation product of phenol. While phenol is mainly obtained from cumene from fossil resources, it has also been produced industrially using forest residues and agriculture wastes taking advantage of the lignin fraction.^9^ The synthesis of phenol using lignin represents a promising pathway but it is also challenging due to the presence of alkyl groups.^10^ However, in the last years, notable advancements have been carried out in this field.^11^ For example, recently, an integrated biorefinery process was devised where 20 wt % of lignin is converted in phenol. In this process, wood birch undergoes reductive catalytic fractionation, resulting in a carbohydrate pulp, suitable for bioethanol production and lignin oil extraction. Subsequently, the phenolic monomers present in the lignin oil are selectively transformed into phenol through gas-phase hydroprocessing and dealkylation reaction.^12^ In another reporting case, phenol has been obtained from lignin by a multistep methodology based in oxidation, decarboxylation, and hydrogenolysis reactions.^10^ Thus, CHO can be considered as a lignin-derived platform molecule with potential for the production of biojet fuel precursors. The proposed catalytic route must necessarily include an increase in the carbon-chain length to meet the required range for aviation fuel (C_9_–C_15_). In this way, CHO can be transformed via C–C coupling through a solventless self-aldol condensation into a C_12_ oxygenated aldol adduct, which should be further hydrodeoxygenated (HDO reaction) to reach the final hydrocarbon product, linear, and/or cyclic. This chemical route starting form cyclic ketones has been recently shown as an alternative approach for producing jet fuel-range cyclic hydrocarbons.^13^ However, the monocondensed C_12_ product can overreact with further CHO molecules to provide larger polycyclic oxygenated alkanes (C_18_–C_24_), no longer suitable for jet fuel purposes.^14^ Therefore, the control of the selectivity toward the monocondensed product appears as a key parameter. The aldol condensation of CHO can be acid or base catalyzed, and acid catalysts like sulfuric acid,^15^ zeolites,^16^ heteropoly acids,^14^ or ion exchange resins^17^ have been used, whereas sodium hydroxide^18^ or hydrotalcite^19^ are examples of base catalysts. In general, basic catalysts often require elevated temperature or long reaction time to obtain an acceptable conversion value.^20^ However, under these conditions, the formation of larger polycyclic alkanes, nonvalid as a suitable jet fuel precursor, are favored. Within acid catalysts, both Brønsted and Lewis acid centers can catalyze aldol condensation, but their mechanisms are different. In industry and academia, Lewis acid catalysts are currently mostly used,^21^ so less research has been carried out using Brønsted acid catalysts for this type of reaction.

During the last decades, mesostructured silica materials have been widely used in different applications.^22−28^ However, the most common usage is as supports for active phases in heterogeneous catalysis. The use of different structure directing agents, or the addition of swelling agents during the synthesis procedure of these materials, allows us to tune the morphology, structure, and pore size of the resultant mesoporous silica.^29^ The incorporation of catalytic functionalities can be carried out simultaneously to the formation of the siliceous mesostructure, through the so-called direct synthesis or co-condensation process, or using postsynthetic chemistry for the functionalization of a previously formed silica structure.^30^ The surface modification of siliceous supports can generate, among others, base or acidic sites on the materials surface. Acid functionalization of mesostructured silicas has been widely reported,^31^ in an attempt to heterogenize the toxic and corrosive mineral acids typically used in homogeneous acid catalysis.^32^ Particularly, in recent years, many studies have been published on the incorporation of sulfonic acid groups onto mesostructured siliceous materials.^33−37^ Moreover, the acid strength of the incorporated SO_3_H group can be tuned by properly selecting the surrounding hydrocarbon moiety, such as propylsulfonic,^38^ arylsulfonic,^39^ or perfluorosulfonic acid moieties,^40^ which widens the range of applications.

The purpose of this work is to evaluate the influence of the pore size and structure of several sulfonic acid-modified mesoporous silica materials in the solventless self-aldol condensation of CHO, aiming at a selective and efficient production of the monocondensed adduct. The structure properties of the raw silica materials are pivotal to determine the incorporation and distribution of acid sites and therefore to their catalytic behavior. Likewise, the textural characteristics of the supports are decisive in terms of catalyst reutilization, which is mandatory in the development of heterogeneous catalysts.

## 2 Experimental Section

### 2.1 Materials

Chemicals were acquired from Merck except for 2-(4-chlorosulfonylphenyl)ethyl-trichlorosilane that was purchased from Abcr, and in all cases, they were used as received. Two commercial polystyrene sulfonic acid materials (Amberlyst-15 and Amberlyst-70) were supplied by Sigma-Aldrich and used as reference catalysts.

### 2.2 Catalyst Preparation

The synthesis procedure of the analyzed silica supports (SBA-3, SBA-15, LP-SBA-15, SBA-16, FDU-12, and SiNF) was based on previous published methods. SBA-3 material was synthesized according to the procedure previously described.^41^ In a typical synthesis, cetyltrimethylammonium bromide (CTAB, 1.96 g) was dissolved in a mixture of HCl (37 wt %, 34.1 mL) and water (79.3 mL) at room temperature. Then, tetraethyl orthosilicate (TEOS, 10 mL), as a silica source, was added dropwise under vigorous agitation, and the mixture was stirred for 3 h. Thereafter, the solid was filtered, washed with water, and dried for 12 h at room temperature. Finally, the surfactant was removed by calcination at 550 °C for 5 h (1.8 °C/min heating ramp). SBA-15 mesoporous silica was synthesized following the classic procedure,^42^ by dissolving at a room-temperature Pluronic P-123 triblock copolymer (8.0 g) in 250 mL of an aqueous solution of HCl (1.9 M). Once dissolved, the temperature was raised to 40 °C, and TEOS (16 g) was added dropwise. After 20 h under strong agitation, the mixture was transferred to a polypropylene bottle and heated at 100 °C under static conditions for 24 h. Finally, the solid was separated by filtration, and the structure-directing agent was eliminated by calcination as for the previous material. For the synthesis of the large-pore variant, the material LP-SBA-15, the procedure was used, as previously described:^43^ Pluronic P-123 (2.4 g) and NH_4_F (0.027 g) were dissolved in an aq. HCl solution (2 M, 55 mL) at an initial temperature of 17 °C in a cold thermostatic bath. After 1 h, a mixture of TEOS (5.5 mL) as the silica source and 1,3,5-triisopropylbenzene (TIPB, 1.2 mL) as the micelle expanding agent was added and stirred for 20 h at the same temperature. Afterward, the mixture was placed into a closed Teflon-lined autoclave and heated in an oven set at 130 °C for 10 h. The solid was then recovered, and the surfactant was removed by calcination as for the previous materials. For the material SBA-16, triblock copolymer Pluronic F-127 (2.7 g) was dissolved in a solution of HCl (5.69 g, 37 wt %) and water (128 g) under agitation.^44^ Then, butanol (8 g) was added, and stirring was continued for 1 h. Next, TEOS (12 g) was added dropwise under stirring for 24 h at 45 °C. The resultant mixture was aged for 24 h at 100 °C. Finally, the solid was recovered and dried, and the surfactant was removed by calcination as for the previous materials. The FDU-12 silica support was synthesized following a previous work,^45^ where Pluronic P-127 (1.0 g) and KCl (5.0 g) were dissolved in aq. HCl (2 M, 61 mL), and the mixture was stirred for 30 min. Next, the micelle swelling agent (TIPB, 1.4 g) was added, and the resultant mixture was stirred for 30 min. Finally, the silica source (TEOS, 4.5 g) was added dropwise. After 3 h of constant stirring, the product was placed in a closed Teflon-lined autoclave and heated under static conditions (130 °C and 4.5 h). The solid was recovered, dried, and calcined as for previous materials. Silica nanoflowers (SiNFs) were prepared following a reported procedure,^46^ by dissolving surfactant CTAB (1.0 g), *n*-butanol (1.0 g), and cyclohexane (12 g) in a aqueous solution of urea (0.4 M, 30 g). Afterward, the silica source TEOS (2.0 g) was added under vigorous agitation at 70 °C for 20 h. The solid was then recovered, washed with ethanol, and dried, and the surfactant was removed by calcination as for the previous materials.

**Figure 1:**
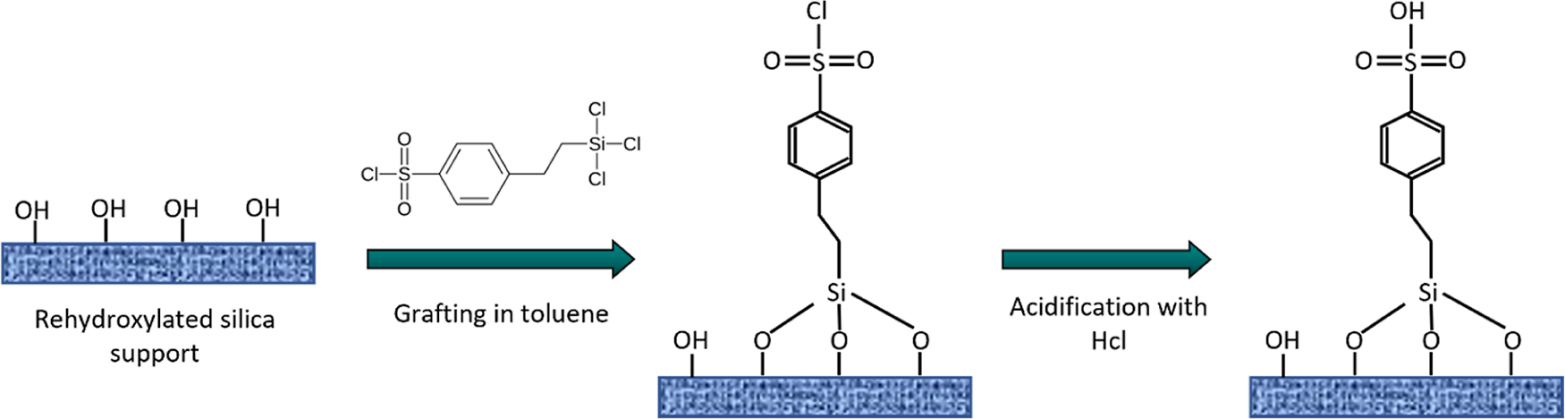
Scheme of the method used for the functionalization of synthesized silica supports with arylsulfonic acid moieties.

After the synthesis of the silica supports and with the objective of increasing the number of silanol groups on the silica surface, a rehydroxylation procedure was carried out. This procedure was previously demonstrated to be effective in increasing the surface silanol concentration in mesoporous silica materials while keeping the mesoscopic order.^27^ For this purpose, a sample of the corresponding silica support (0.5 g) was suspended in aq. HCl (1 M, 50 mL) and heated under reflux for 4 h. After this time, the solid was recovered, and the remaining acid was washed off with water until neutral pH. Surface functionalization of the activated silica supports with arylsulfonic acid groups was performed following a postsynthesis grafting procedure using 2-(4-chlorosulfonylphenyl)ethyl-trichlorosilane (0.7 g) as a sulfur precursor in dry toluene under a nitrogen atmosphere for 24 h at 110 °C. The resultant materials were recovered by filtration and thoroughly washed with fresh toluene and dried. Finally, acidification of the –SO_2_Cl moieties was accomplished by acid exchange in aq. HCl (1 M) to obtain the acid species (−SO_3_H) (Figure 1). The resultant sulfonic-acid-functionalized silica materials were denoted as SO_3_H-[*name of the support*].

### 2.3 Catalyst Characterization

X-ray diffraction (XRD) patterns were acquired on a Philips X́PERT diffractometer by using Cu Kα radiation. Data were recorded from 0.6 to 5° (2θ) with a resolution of 0.02°. Transmission electron microscopy (TEM) microphotographs were obtained on a JEM-1400 instrument operated at 120 kV. Nitrogen adsorption and desorption isotherms were measured at 77 K using a Micromeritics Tristar II analyzer. The samples were previously outgassed at 120 °C overnight. The isotherms were analyzed using the BET method to determine the specific surface area, pore sizes distributions were calculated through the BJH method using the KJS correction, and total pore volume was taken at *P*/*P*_0_ = 0.975. Sulfur content analyses were carried out in a Flash 2000 Organic Elemental Analyzer apparatus. Thermogravimetric analyses were performed in a SDT 2960 equipment from TA Instruments using 5 °C/min ramp up to 900 °C in the air atmosphere.

### 2.4 Reaction Procedure

The solventless self-condensation of CHO was carried out in a 100 mL glass round-bottom flask under temperature control and continuous magnetic stirring. In a typical catalytic run, the reaction mixture consisted of 30.0 g of CHO, 0.6 g of sulfolane (as internal standard), and 0.3 g of catalyst. The reaction temperature range was established in preliminary experiments using the commercial Amberlyst-15 catalysts, in the interval 80–120 °C, while the reaction time was evaluated up to 24 h. Reaction samples were analyzed by gas chromatography using Agilent 9000 equipment with a DB-5 column (30 m × 0.32 mm, DF = 0.25 μm) and a flame ionization detector. Injector and detector temperatures were 270 and 300 °C, respectively, and the final column temperature reached 270 °C. CHO and dimer adduct quantification was based on the calibration of the commercially available products using sulfolane as the internal standard. Catalytic results are displayed in terms of conversion of CHO (*X*_CHO_) and selectivity (*S*_DM_) toward the two identified dimers, denoted as DMI and DMII, as shown below in Figure 4 (eqs 1 and 2).
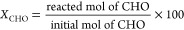
1
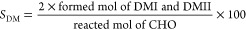
2

## 3 Results and Discussion

### 3.1 Characterization

**Figure 2:**
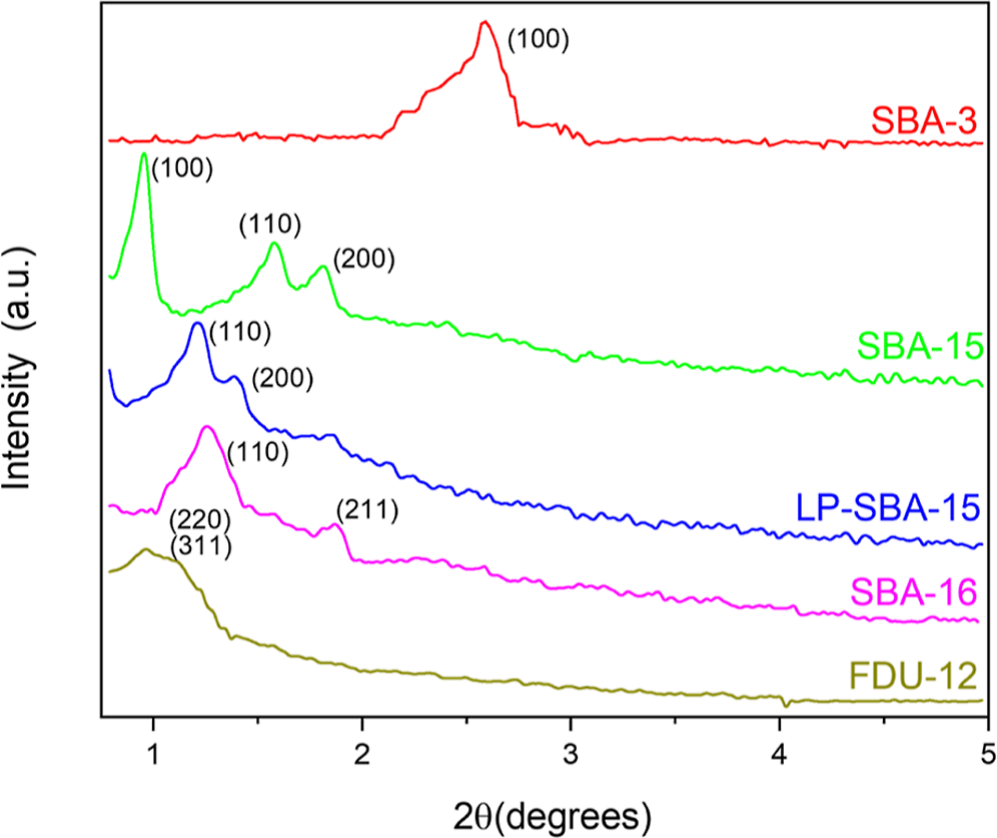
Powder XRD patterns of the synthesized mesoporous silica supports.

Figure 2 shows the powder XRD patterns of the synthesized silica supports. SBA-3, SBA-15, and LP-SBA-15 mesoporous materials exhibit the characteristic diffraction pattern of hexagonal *p*6*mm* symmetry.^42^ However, among these materials, a variation in the 2-theta angle of the (100) diffraction peak is observed due to the difference in the unit cell size, with a reduction in the angle value when the unit cell size increases.^47^ SBA-16 sample shows the diffraction peaks (110) and (200) typical of cubic *Im*3*m* symmetry,^48^ while FDU-12 silica displays the diffraction peaks (220) and (311) attributed to *Fm*3*m* symmetry.^49^ SiNF material is not displayed in this figure because the nanoflower configuration, with silica branches extending from a central core, does not present any regular structural symmetry (amorphous pattern).

In order to confirm the structure and pore morphology of the synthesized silica supports, TEM images were obtained (Figure S1). The micrographs depicted in Figure S1a–c show the ordered hexagonal structure corresponding to SBA-3, SBA-15, and LP-SBA-15, respectively. These images confirm the XRD data, showing the typical 2D hexagonal structures of pore arrays with different lattice distances between pore axes. As expected, the larger pore size corresponds to the expanded large-pore LP-SBA-15 material, whereas the smaller pore size is shown by the SBA-3 silica material. This series of pore-increasing hexagonal supports will allow us to analyze the effect of the pore size on both the organic functionalization with –SO_3_H groups and the catalytic performance. For SBA-16 and FDU-12 materials (Figure S1d,e, respectively), an ordered cubic structure can be observed, in agreement with the XRD patterns. Finally, Figure S1f–h shows the micrographs of the SiNF sample with an open framework of tortuous radial channels from the core to the spherical surface, like those previously reported.^46^ Noteworthy, the homogeneous particle size of this support is by far the smallest among the series of silica supports (spheres around 100–200 nm in diameter).

**Figure 3:**
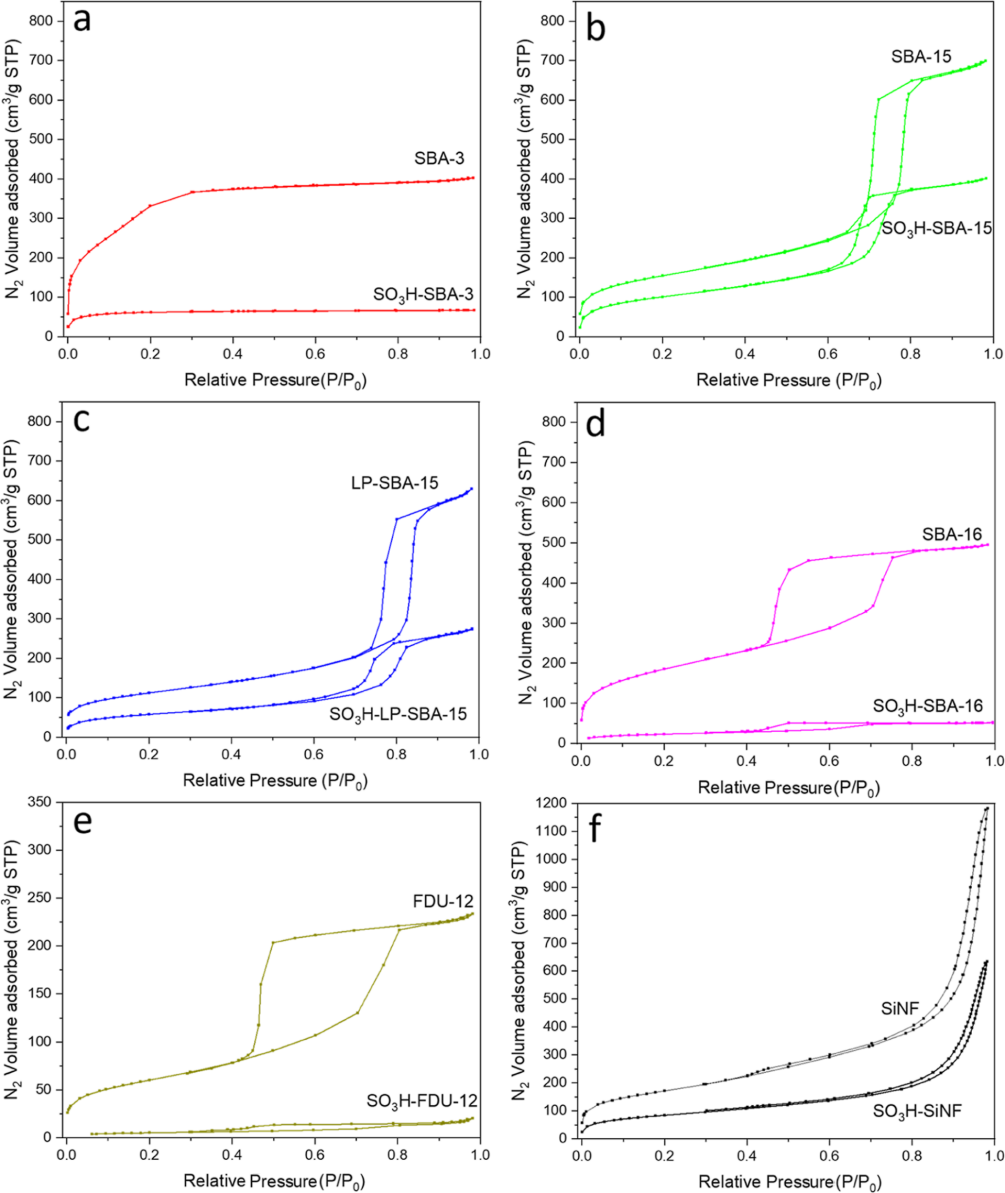
N_2_ adsorption–desorption isotherms of pure silica supports and the corresponding sulfonic acid-functionalized materials: (a) SBA-3 and SO_3_H-SBA-3, (b) SBA-15 and SO_3_H-SBA-15, (c) LP-SBA-15 and SO_3_H-LP-SBA-15, (d) SBA-16 and SO_3_H-SBA-16, (e) FDU-12 and SO_3_H-FDU-12, and (f) SiNF and SO_3_H–SiNF.

Figure 3 represents the N_2_ adsorption–desorption isotherms of the silica supports and the corresponding SO_3_H-functionalized materials. The isotherm for the SBA-3 silica sample (Figure 3a) displays a superposition of type-I (for micropores) and type-IV (for mesopores) isotherms according to the IUPAC classification^50,51^ due to the presence of both types of porosity inside of structure. The isotherms corresponding to SBA-15 and LP-SBA-15 silica materials (Figure 3b,c), while presenting also some microporosity as a consequence of the removal of hydrophilic poly(ethylene oxide) chains of the triblock copolymer template occluded within the siliceous walls, which is typical in SBA-type materials with hexagonal structure,^52^ mainly correspond to type-IV isotherms displaying well-defined H1 hysteresis loops. The higher relative pressure of capillary condensation in the LP-SBA-15 material is attributed to a structure with enlarged pore diameter. SBA-16 isotherm (Figure 3d) shows a H2-type hysteresis loop, typical of materials with 3D pore-network connectivity, while for FDU-12 sample (Figure 3e), a broad hysteresis loop can be observed owing to the presence of cage-like mesopores with entrances narrower than the cage diameter.^49^ Nanoflower silica, SiNF (Figure 3f), mainly presents interstitial porosity, in the relative pressure range of 0.8–1.0, which is attributed to the spaces between silica branches in the nanoflower configuration. Also, a small contribution of type-IV isotherm with hysteresis loop in the range 0.4–0.6 can be observed in this material, indicating the presence of some mesoporosity in agreement with the TEM image (Figure S1).

With the incorporation by grafting of aryl sulfonic groups into the mesoporous silica structures, a remarkable decrease in the adsorbed nitrogen volume is observed for all catalysts due to the high degree of functionality incorporated. However, this behavior is different, depending on the support. Thus, the highest reductions are displayed by SO_3_H-SBA-3, SO_3_H-SBA-16, and SO_3_H-FDU-12 materials (Figure 3a,d,e), with a diminution in the adsorbed N_2_ volume close to 90%. In the case of SO_3_H-SBA-3, this fact is attributed to the relatively small pore size shown by this material (Table 1), suggesting saturation of the mesoporous structure after functionalization with aryl-sulfonic acid moieties. For the cubic structures, SO_3_H-SBA-16 and SO_3_H-FDU-12, the high reduction in the nitrogen adsorption capacity upon functionalization can be ascribed to the saturation of the entrances that connect the spherical cage-like mesopores since such entrances are narrower than the cage diameter.^49^ The SBA-15-type materials, as well as the nonordered SiNF sample, are able to retain a higher degree of porosity after surface modification with –SO_3_H moieties. Thus, as can be judged from the almost parallel adsorption/desorption branches in these materials between pure silica and organically modified samples, a significant level of mesoporosity is still maintained. This can be attributed to the larger pore sizes of these samples, which help limit the blocking effect observed in the small pore supports.

**Table 1:** Textural and Acid Properties of the Synthesized Silica Materials

catalyst	*S*_BET_ (m^2^·g^–1^)	*V*_p_^a^(cm^3^·g^–x1^)	Dp^a^(Å)	S content^b^ (mmol S/g)	S concentration^c^ (μmol S/m^2^)
SBA-3	1199	0.67	20		
SO_3_H-SBA-3	195	0.11	18	1.90	9.7
SBA-15	557	1.05	89		
SO_3_H-SBA-15	370	0.60	78	1.01	2.7
LP-SBA-15	405	0.94	128		
SO_3_H-LP-SBA-15	211	0.40	108	0.88	4.2
SBA-16	675	0.73	75		
SO_3_H-SBA-16	85	0.08	58	2.10	24.7
FDU-12	250	0.35	85		
SO_3_H-FDU-12	50	0.03	79	1.10	22.0
SiNF	618	1.17	meso-macro		
SO_3_H–SiNF	301	0.65	meso-macro	0.77	2.6
Amberlyst-15^d^	45		macro	4.80	106.7
Amberlyst-70^d^	36		macro	2.55	70.8

a Total pore volume and pore size calculated by the BJH method from the adsorption branch of the corresponding nitrogen isotherm.

b Sulfur content calculated from elemental analysis.

c Ratio between S content and BET surface values.

d Characterization data as supplied by the manufacturer.

Table 1 summarizes the textural and acidic properties of the synthesized materials. As textural properties, specific surface area, pore volume, and mean pore diameter are included, while as acid properties, the sulfur content and sulfur surface concentration, calculated from elemental analysis, are incorporated. As a result of the different synthesis conditions and surfactants employed, purely silica supports with a wide range of textural properties were obtained, with mean pore diameter values ranging from 20 Å for SBA-3 sample up to 128 Å for LP-SBA-15 material. In this last case, the use of an organic swelling agent during the synthesis procedure causes a notable increase in pore size with respect to the conventional SBA-15 sample (128 vs 89 Å). Regarding the textural properties of the functionalized materials, a notable reduction in specific surface area and pore volume is observed in all the materials, which brings to light the success of the grafting procedure. As previously discussed, the highest reduction of the textural properties is shown by small-pore and cubic materials: SO_3_H-SBA-3, SO_3_H-SBA-16, and SO_3_H-FDU-12 materials. However, it must be noted that SO_3_H-SBA-3 and SO_3_H-SBA-16 samples still display a BET surface of 195 and 85 m^2^/g with pore volumes of 0.11 and 0.08 cm^3^/g, respectively. As regards to the other sulfonic acid-modified samples, they display adequate textural parameters, still well within the mesoporous range, with specific surface area and pore volume between 370 m^2^/g of 0.60 cm^3^/g for SO_3_H-SBA-15 material, and 211 m^2^/g and 0.40 cm^3^/g for SO_3_H-LP-SBA-15 sample. Therefore, these acid-modified materials are expected to perform well in catalytic applications based on the conversion of relatively bulky products like the autocondensation adduct of CHO herein analyzed.

With respect to the acid properties, the sulfur content ranges from 2.10 to 0.77 mmol of S/g depending on the silica support, indicating a high degree of organic incorporation, even over the typical range of sulfonic mesoporous catalysts. This would be in consonance with the marked effect on the textural properties. Among the mesoporous silica supports, a nonlineal correlation can be established between a higher surface area of silica support before grating and the S loading in the materials after grafting. This is consistent with the postsynthetic surface grafting methodology used for the functionalization of the materials, which depends mainly on the available surface silanols. However, the SiNF material (SO_3_H–SiNF) does not follow this trend. This exception can be explained in terms of the nonordered external nature of the porosity in this material. The parameter of sulfur surface concentration, calculated as μmol of S per square meter of the BET surface area, is also included in Table 1. In this case, the materials with cubic structures (SO_3_H-SBA-16 and SO_3_H-FDU-12) present the highest S concentration values (>20 μmol S/m^2^), corroborating the increased probability of partial mesopore blocking in these materials. On the other hand, the supports with more open porous structure and larger pore size, like SO_3_H-SBA-15, SO_3_H-LP-SBA-15, and SO_3_H–SiNF, exhibit a homogeneous acid center distribution, in the range 2.6–4.2 μmol S/m^2^. Finally, for comparison purposes in the following catalytic evaluation, BET surface area and acid capacity corresponding to commercial heterogeneous catalysts Ambelyst-15 and Amberlys-70 are also included in Table 1. These acid resins are based on sulfonated polystyrene-based polymers with a high loading of sulfonic acid centers (4.80 and 2.55 mmol S/g, respectively). It should be noted that these selected commercial catalysts show values of sulfonic groups concentration very higher than the rest of the samples.

### 3.2 Assessment of the Catalytic Activity

**Figure 4:**
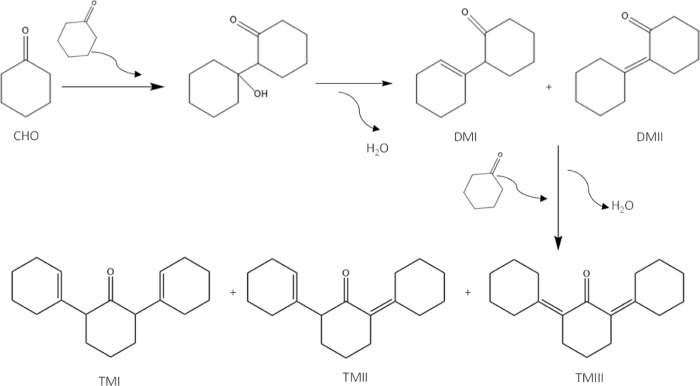
Reaction scheme for the CHO self-condensation reaction.^18^

The reaction of biomass-derived cyclic ketones such as CHO to monocondensed products has an enormous potential to obtain precursors for the synthesis of renewable jet fuel. Therefore, the catalytic activity of the sulfonic-modified silica materials was evaluated in the solventless self-aldol condensation of CHO. The main product of this aldol condensation consists of an isomeric mixture of CHO dimers, namely, 2-(1-cyclohexen-1-yl)-cyclohexanone (dimer 1 or DMI) and cyclohexylidene-cyclohexanone (dimer 2 or DMII) (Figure 4). It has been reported that the ratio of DMI/DMII is typically about 9:1 over heterogeneous acid catalysts.^16^ It must be noted that an intermediate is first formed during the condensation, the hydroxylated adduct 1-hydroxy-[1,1-bicyclohexyl]-2-one, but in the presence of strong Brønsted acid sites, is rapidly dehydrated to yield the isomeric mixture. Furthermore, the dimers can further react with other ketone via aldol condensation to yield heavy cyclic compounds (TMI, TMII, and TMIII), too large to be appropriate for jet fuel blending.^14^ Therefore, for this reaction, the catalyst and the selected reaction conditions play an important role to get a high selectivity toward the monocondensed products (dimers).

In this study, the reaction conditions were preliminarily explored using the commercial sulfonic acid resin Amberlyst-15 as a catalyst. The range of temperatures (80–120 °C) and the reaction time (up to 24 h) were chosen based on previous studies,^53,54^ with the objective to keep the dimers selectivity close to 100% while obtaining high CHO conversion. As a result (Table S1), a temperature of 100 °C was chosen to evaluate the catalytic activity of the synthesized sulfonic acid-modified silica materials since higher temperature (120 °C), though leading to magnified CHO conversion, also implied a significant loss of selectivity to the monocondensed products. In the same line, catalyst loading was fixed at 1% (w/w) based on CHO mass. On the other hand, to rule out the influence of the thermal effect on the self-condensation of CHO, a blank test was carried out (100 °C, 24 h), leading to a complete absence of condensation activity.

**Figure 5:**
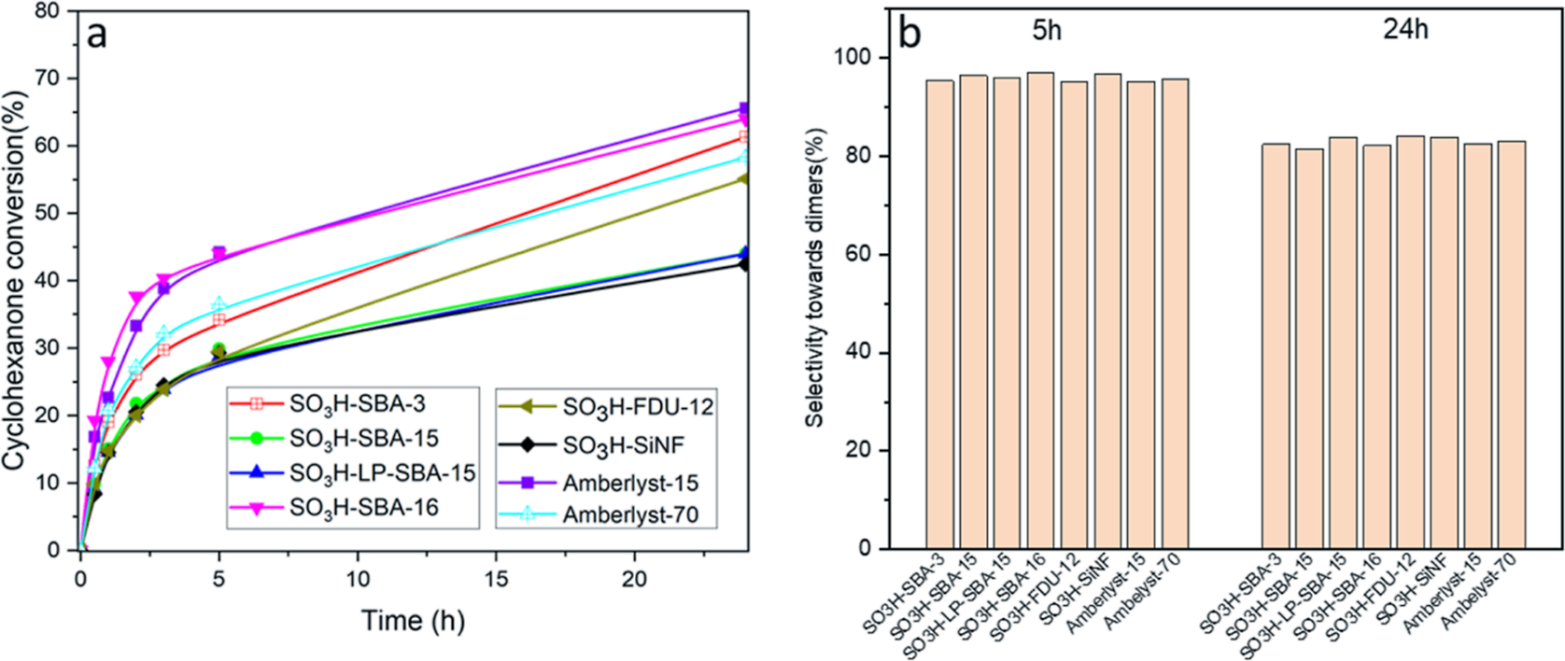
CHO conversion (a) and selectivity toward the two dimers (b) of the different sulfonic acid-modified materials. Temperature, 100 °C; catalyst loading:1 wt % based on CHO mass; and solventless conditions.

Figure 5 displays the catalytic results of the sulfonic-modified silica samples and the reference commercial heterogeneous catalysts (Amberlyst-15 and Amberlyst-70), in terms of conversion of CHO (a) and selectivity toward the dimers DMI and DMII (b). Noteworthy, the selectivity to the monocondensed products remains >95% even at after 5 h of reaction, irrespective of the catalyst. The presence of undesired heavy polycyclic adducts only becomes significant at the longer analyzed reaction time (24 h), when the selectivity to dimers comes down to values in the range 80–85%. Therefore, under these reaction conditions, there is no appreciable effect of the catalyst structure on the selectivity. To confirm this conclusion, conversion-selectivity values were plotted for 5 and 24 h of reaction (Figure S2), demonstrating that there is not a trend between the different synthesized materials. This behavior can be attributed to the presence of the identical sulfonic acid centers in all the materials, and to the high inherent selectivity of the reaction itself, where the variations in the catalytic performance are ascribed to the concentration of aryl-sulfonic groups and their accessibility. On the other hand, the reaction rate decreases with the reaction time for all the materials, especially at long reaction times (>5 h). This fact can be attributed to a strong adsorption of the generated water on the catalyst active centers, which are highly hygroscopic.^17^

**Figure 6:**
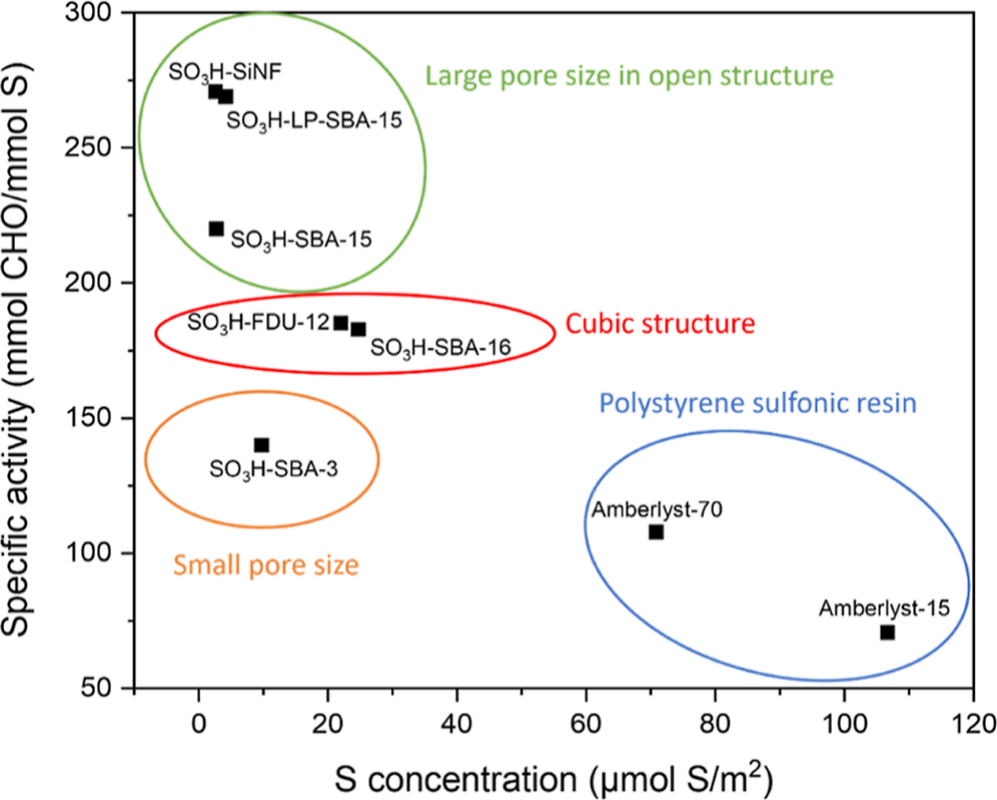
Specific activity after 2 h of reaction time vs sulfur surface concentration for the different sulfonic acid-modified catalysts. Temperature, 100 °C; catalyst loading: 1 wt % based on CHO mass; and solventless conditions.

Correlating the activity with the acid properties of the catalysts, CHO conversion clearly increases with the loading of sulfonic acid groups in the catalyst. Thus, materials with high sulfur content (both Amberlyst resins, SO_3_H-SBA-3, SO_3_H-SBA-16, and SO_3_H-FDU-12) exhibit higher conversion rates, while the catalysts with lower number of acid centers (SO_3_H-SBA-15, SO_3_H-LP-SBA-15, and SO_3_H–SiNF) yield lower CHO conversion rates. However, there is not a proportional ratio between sulfur content and CHO conversion since Amberlyst-15 and SO_3_H-SBA-16 display a similar catalytic performance when the commercial resin presents a higher concentration of sulfonic acid sites (Table 1). A similar behavior occurs with Amberlyst-70 and SO_3_H-SBA-3. Hence, a more precise analysis of the catalytic behavior should be done to evaluate the effect of the structure, textural properties, and the sulfonic acid surface density of each catalyst. In this way, Figure 6 compares the specific activity, after just 2 h of reaction to minimize the influence of water adsorption phenomena on the catalytic results, with the S surface density, as defined in Table 1. The specific activity has been calculated as the millimoles of reacted CHO per mmol S at a given reaction time.

As evidenced in the plot, catalysts with a lower sulfur concentration exhibit a higher specific activity, while materials with a high sulfur content dramatically reduce their activity per acid center. This behavior can be explained with reference to the nature, structure, and textural properties of each support. The commercial polystyrene-based sulfonic resins combine high sulfonic groups loadings (4.80 and 2.55 mmol S/g for Amberlyst-15 and 70, respectively) with reduced surface areas (45 and 36 m^2^/g), resulting in a high surface density of SO_3_H groups. In ketone aldol condensation reactions, it has been reported that the distance between neighboring acid centers plays an important role in their activity. Thus, the nucleophilic addition from the activated enol group onto adsorbed ketone requires a minimum distance between vicinal acid groups to proceed adequately.^55,56^ However, the high acid center concentration over nonordered structure in Amberlyst catalysts seems to produce a strong competition among the –SO_3_H groups for CHO molecule chemisorption, so that part of the acid center necessarily remains catalytically unavailable. Furthermore, a high concentration of acid sites also helps in increasing the hydrophilicity of the catalyst surface, facilitating the detrimental accumulation of water molecules surrounding the active sites.^57^

For the SO_3_H-SBA-16, SO_3_H-FDU-12, and SO_3_H-SBA-3 catalysts, despite their lower sulfur concentrations as compared to the resins, the specific catalytic activity is still limited. This can be explained in terms of partial inaccessibility of CHO molecules to the sulfonic sites caused by the blockage of the mesopore entrances upon the surface anchoring procedure carried out to incorporate the acid function. As previously discussed, such a partial blockage is attributed to the small pore size in the case of SBA-3-type catalyst, and to a bottleneck effect as a consequence of the small size of the pore entrances interconnecting the cubic structures conforming the SBA-16 and FDU-12 materials.

On the other hand, the catalysts with the lowest S surface concentrations (SO_3_H-SBA-15, SO_3_H-LP-SBA-15, and SO_3_H–SiNF) show the best catalytic performance in terms of specific activity per acid site. These materials display open structures with large pores, enabling an optimal surface distribution of anchored aryl-sulfonic acid groups, which maximizes their intrinsic activity (reaching values > 200 mmol CHO/mmol S). Moreover, such open pore structures are also beneficial for the diffusion of CHO molecules and condensation products through the porous structure.^40^ This is especially the case for the samples SO_3_H-LP-SBA-15 and SO_3_H–SiNF, which show the largest pore sizes.

### 3.3 Reusability of Synthesized Catalysts

**Figure 7:**
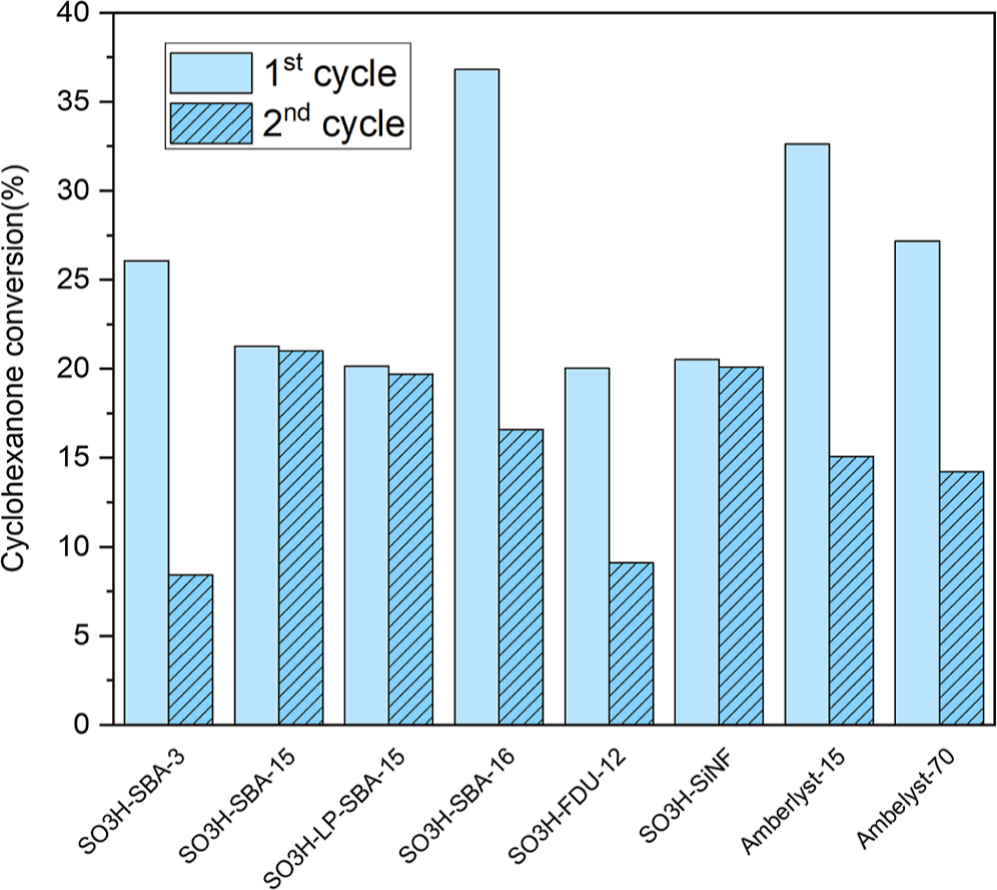
Conversion of CHO over the different sulfonic-modified materials in two consecutive reaction cycles. Reaction conditions: reaction time: 2 h, temperature: 100 °C, catalyst loading: 1 wt % based on CHO mass, and solventless conditions.

The reusability of the evaluated materials was studied under the same reaction conditions (solventless, 100 °C, 1 wt % catalyst loading), fixing the reaction time at 2 h to avoid saturation of the system. After the first reaction cycle, the catalysts were filtered off from the reaction media and simply washed at room temperature with a mixture ethanol-acetone (1:1 vol) for 30 min to remove entrapped reactants and products. The washed solids were dried at 60 °C under vacuum and subsequently used in a second reaction cycle under otherwise identical conditions. Figure 7 shows the results in terms of CHO conversion (the selectivity of the monocondensed products remains >95% for all the samples) in both catalytic runs. As shown, the commercial resins Amberlyst-15 and Amberlyst-70, as well as the synthesized SO_3_H-SBA-16, SO_3_H-FDU-12, and SO_3_H-SBA-3 materials, clearly evidence a loss of activity, >50% of the initial CHO conversion. In contrast, SO_3_H-SBA-15, SO_3_H-LP-SBA-15, and SO_3_H–SiNF yielded similar CHO conversion values in the second reaction cycle, evidencing no significant activity loss. Therefore, there is a clear correlation between the reusability of the materials and the specific activity values reported in Figure 6, indicating that high S surface concentrations and small pores enhance the deactivation of these types of acid catalysts.

**Figure 8:**
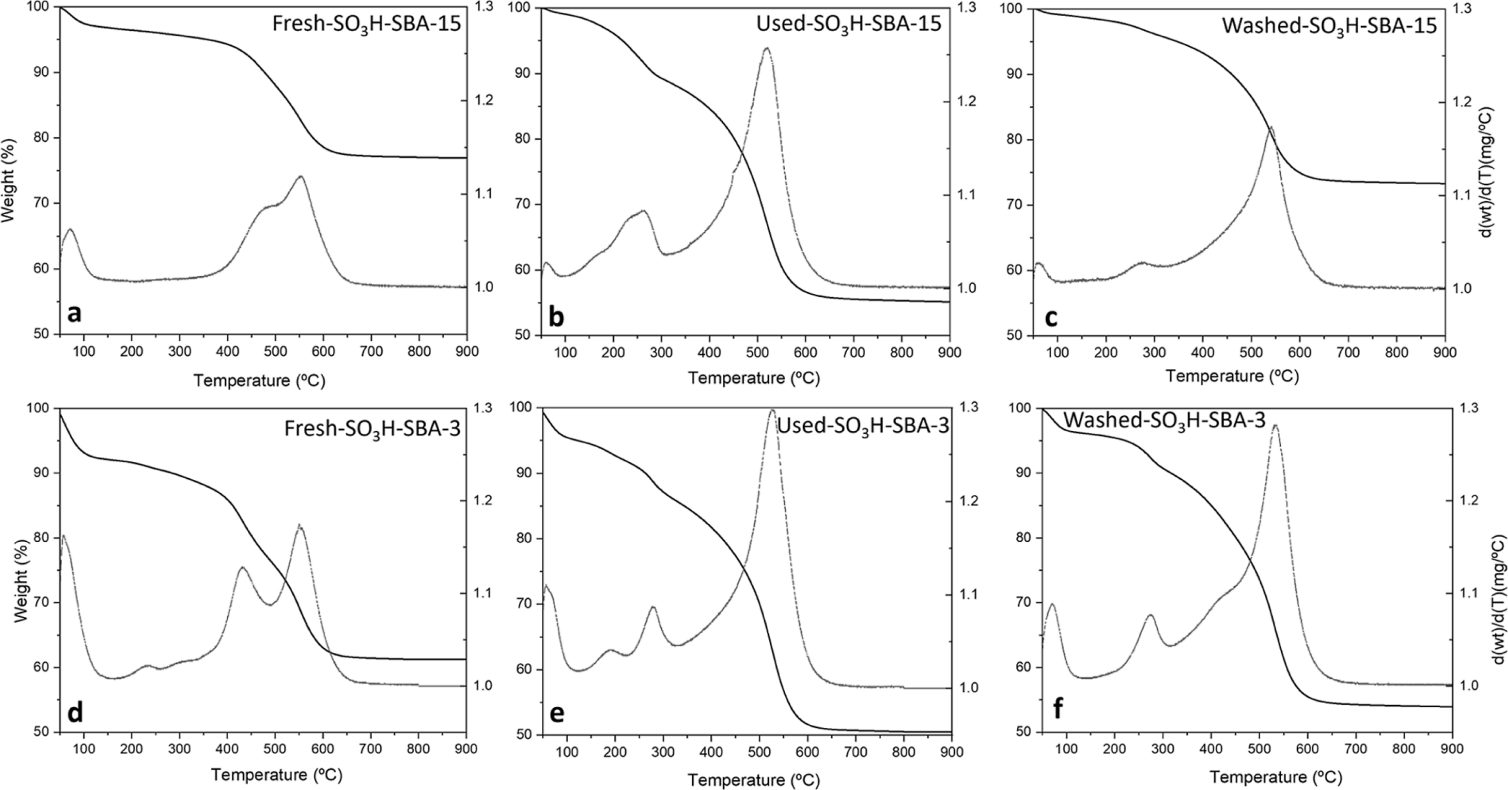
TGA analysis of fresh and spent (after the first reaction cycle) catalysts and after the washing treatment for SO_3_H-SBA-15 and SO_3_H-SBA-3.

To analyze the deactivation causes, the possibility of leaching of SO_3_H groups was assessed by analyzing the sulfur content of the spent catalysts. However, the elemental analysis verified that S content remained constant before and after reaction, though evidencing an increase of the C content for all the catalysts. Therefore, fouling of catalysts was also evaluated, and thermogravimetric analysis (TGA) before and after the first reaction, as well as after washing treatment, were carried out for SO_3_H-SBA-15 and SO_3_H-SBA-3 samples, as representatives of catalysts with good and poor reusability, respectively (Figure 8). The TGA corresponding to the fresh materials (Figure 8a,d) shows a first small weight loss below 100 °C that can be attributed to ambient humidity and toluene residue from the grafting procedure. The main weight loss, between 350 and 700 °C, corresponds to the thermal decomposition of the anchored aryl-sulfonic acid groups. This loss is quantified in 17.9 wt % in the case of SO_3_H-SBA-15 catalyst and 27.0 wt % for SO_3_H-SBA-3, in agreement with the S content obtained via elemental analysis (Table 1). In the spent catalysts (Figure 8b,e), a similar new weight loss can be observed between 100 and 350 °C, which is attributed to adsorbed CHO and condensation products within the catalyst structure. However, in the washed materials (Figure 8c,f), there is a significant difference in the amount of adsorbed products for this temperature range (100–350 °C), being higher for SBA-3-type material (8.1 wt %) than that for the SBA-15-type one (3.1 wt %). This fact confirms the better diffusion properties in the SO_3_H-SBA-15 catalyst owing to its higher pore size and open structure. In turn, in the SO_3_H-SBA-3 catalyst, the washing treatment is not sufficient to remove the reactants and products embedded in the structure, leading to a reduced performance in the second catalytic run (Figure 7).

**Figure 9:**
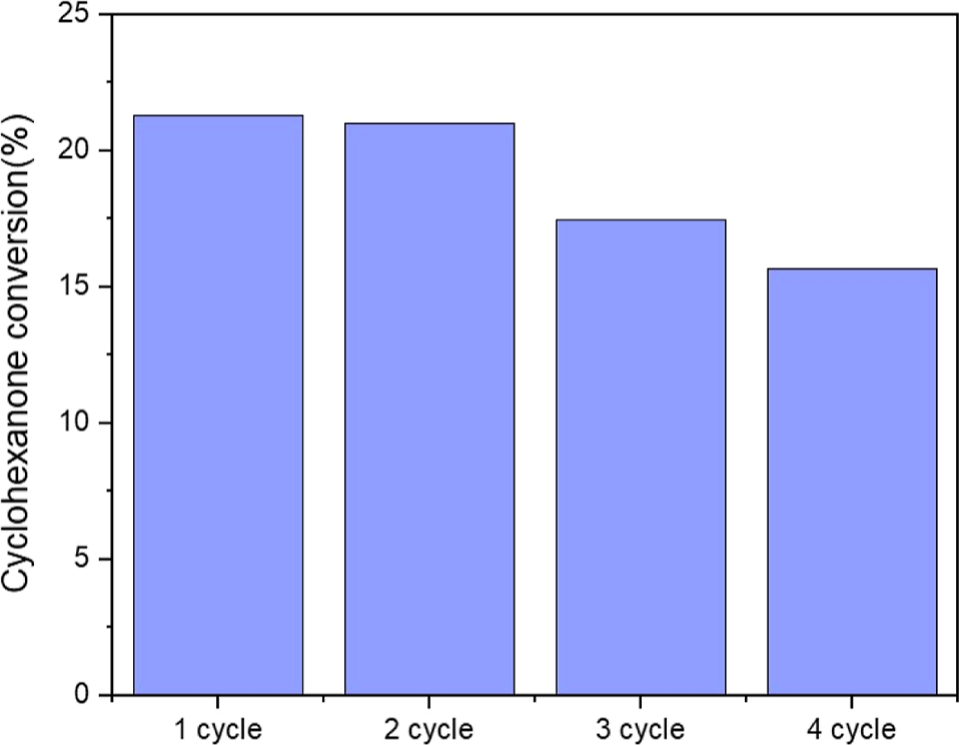
Conversion of CHO using SO_3_H-SBA-15 as the catalyst in four successive reaction cycles. Reaction conditions: time: 2 h, temperature: 100 °C, catalyst loading: 1 wt % based on CHO mass, and solventless conditions.

Finally, the reusability of catalyst SO_3_H-SBA-15 after several consecutive reaction cycles was evaluated using the same washing method as in the second cycle (Figure 7) but followed by acid exchange in aq. HCl (1 M) to reactivate the protonated form of the acid sites. As shown in Figure 9, the SO_3_H-SBA-15 material shows a progressive loss of activity in the third and fourth reaction cycle (though keeping the high selectivity values of the fresh catalyst). This fact can be attributed to the adsorption of CHO and condensation products within the catalyst structure. As previously indicated, the washing process of the SO_3_H-SBA-15 spent catalysts allowed the elimination of embedded reaction products, which has been attributed to the better diffusion properties of this material as compared with others. This is evident from the TGA results (Figure 8a–c). However, in the third and fourth cycles, apparently part of the adsorbed compounds is no longer removable, leading to a slight decrease in catalytic activity. Therefore, it is demonstrated that with structures with large pore size and open structure, it is possible to reduce and delay the otherwise unavoidable progressive deactivation of sulfonic-modified catalysts in this type of reaction.

## 4 Conclusions

Six mesoporous silica supports with different textural properties and structures have been successfully prepared and postsynthetically functionalized with aryl-sulfonic acid groups. The organic incorporation onto the silica materials afforded high sulfur contents, ranging from 0.77 to 2.10 mmol S/g, which introduces a significant effect on the textural properties of the supports with lower pore size and cubic structure. The sulfonic acid-modified silica catalysts have been applied in the solventless self-condensation of CHO to obtain biojet fuel precursors. The catalytic results have highlighted the high specific activity of the synthesized materials, superior to reference commercial sulfonic Amberlyst catalysts. SBA-15-type materials and SiNF nanoflower support have shown an optimal surface distribution of anchored aryl-sulfonic acid groups, maximizing their intrinsic activity (>200 mmol CHO/mmol S). Moreover, their open pore structures are also beneficial for the diffusion of CHO molecules and condensation products through the porous structure, minimizing the deactivation of the catalysts for reusing purposes.

## Supporting Information Available

TEM images of the silica supports and additional catalytic results of synthesized and commercial materials (PDF)

## References

[ref1] Churchill J. G. B.; Borugadda V. B.; Dalai A. K. A Review on the Production and Application of Tall Oil with a Focus on Sustainable Fuels. Renewable Sustainable Energy Rev. 2024, 191, 114098 10.1016/j.rser.2023.114098.

[ref2] International Energy Agency Transport, Energy and CO2 - Moving toward Sustainability. Paris, 2009.

[ref3] Simone N. W.; Stettler M. E. J.; Barrett S. R. H. Rapid Estimation of Global Civil Aviation Emissions with Uncertainty Quantification. Transp. Res. D: Transp. Environ. 2013, 25, 33–41. 10.1016/j.trd.2013.07.001.

[ref4] Lim M.; Luckert M. K. M.; Qiu F. Economic Opportunities and Challenges in Biojet Production: A Literature Review and Analysis. Biomass Bioenergy 2023, 170, 106727 10.1016/j.biombioe.2023.106727.

[ref5] European Commission Communication from the Commission to the European Parliament, the Council, the European Economic and Social Committee and the Committee of the Regions: The European Green Deal; Publications Office of the European Union, 2019.

[ref6] European Commission DIRECTIVE (EU) 2018/2001 of the EUROPEAN PARLIAMENT and of the COUNCIL of 11 December 2018 on the Promotion of the Use of Energy from Renewable Sources; European Commission, 2018.

[ref7] Gutiérrez-Antonio C.; Gómez-Castro F.; de Lira-Flores J. A.; Hernández S. A Review on the Production Processes of Renewable Jet Fuel. Renewable Sustainable Energy Rev. 2017, 79, 709–729. 10.1016/j.rser.2017.05.108.

[ref8] Sun J.; Shao S.; Hu X.; Li X.; Zhang H. Synthesis of Oxygen-Containing Precursors of Aviation Fuel via Carbonylation of the Aqueous Bio-Oil Fraction Followed by C-C Coupling. ACS Sustain. Chem. Eng. 2022, 10 (33), 11030–11040. 10.1021/acssuschemeng.2c03379.

[ref9] Li Q.; Nie G.; Wang H.; Zou J.-J.; Yu S.; Yu H.; Jin X.; Zhang D.; Shi H.; Zhao D. Synthesis of High-Grade Jet Fuel Blending Precursors by Aldol Condensation of Lignocellulosic Ketones Using HfTPA/MCM-41 with Strong Acids and Enhanced Stability. Appl. Catal., B 2023, 325, 122330 10.1016/j.apcatb.2022.122330.

[ref10] Wang M.; Liu M.; Li H.; Zhao Z.; Zhang X.; Wang F. Dealkylation of Lignin to Phenol via Oxidation-Hydrogenation Strategy. ACS Catal. 2018, 8 (8), 6837–6843. 10.1021/acscatal.8b00886.

[ref11] Yan J.; Meng Q.; Shen X.; Chen B.; Sun Y.; Xiang J.; Liu H.; Han B. Selective Valorization of Lignin to Phenol by Direct Transformation of C Sp2 -C Sp3 and C-O Bonds. Sci. Adv. 2020, 6 (45), eabd1951 10.1126/sciadv.abd1951. 33158871 PMC7673717

[ref12] Liao Y.; Koelewijn S.-F.; Van den Bossche G.; Van Aelst J.; Van den Bosch S.; Renders T.; Navare K.; Nicolaï T.; Van Aelst K.; Maesen M.; Matsushima H.; Thevelein J. M.; Van Acker K.; Lagrain B.; Verboekend D.; Sels B. F. A Sustainable Wood Biorefinery for Low-Carbon Footprint Chemicals Production. Science 2020, 367 (6484), 1385–1390. 10.1126/science.aau1567. 32054697

[ref13] Gunasekaran V.; Rathinam Y.; Ganesan R.; Gurusamy H. High-Density Jet-Fuel Hydrocarbons from Biomass-Derived Cyclic Ketones via Vapor Phase Hydrodeoxygenation over Ru-Ni 2 P/Al (10)-KIT-6. Energy Fuels 2023, 37 (11), 7881–7903. 10.1021/acs.energyfuels.3c00354.

[ref14] Deng Q.; Nie G.; Pan L.; Zou J.-J.; Zhang X.; Wang L. Highly Selective Self-Condensation of Cyclic Ketones Using MOF-Encapsulating Phosphotungstic Acid for Renewable High-Density Fuel. Green Chem. 2015, 17 (8), 4473–4481. 10.1039/C5GC01287B.

[ref15] Wu T. R.; Su W. C.; Peng Y. S. TW Patent 250145, 2006.

[ref16] Chen Y.; Yuan S.; Yin H.; Chen Z.; Mao C. Kinetics of the Reversible Dimerization Reaction of Cyclohexanone over γ-Alumina Catalyst. React. Kinet., Mech. Catal. 2011, 102 (1), 183–194. 10.1007/s11144-010-0250-7.

[ref17] Aragon J. M.; Vegas J. M. R.; Jodra L. G. Self-Condensation of Cyclohexanone Catalyzed by Amberlyst-15. Study of Diffusional Resistances and Deactivation of the Catalyst. Ind. Eng. Chem. Res. 1994, 33 (3), 592–599. 10.1021/ie00027a016.

[ref18] Lorenzo D.; Santos A.; Simón E.; Romero A. Kinetic of Alkali Catalyzed Self-Condensation of Cyclohexanone. Ind. Eng. Chem. Res. 2013, 52 (6), 2257–2265. 10.1021/ie303213p.

[ref19] Reichle W. T. Catalysts for Aldol Condensations. U.S. Patent 4,458,026 A, 1984.

[ref20] Kekana L.; Bingwa N. Solvent-Free Cross Aldol Condensation of Aldehydes and Ketones over SrMo1-XNixO3-δ Perovskite Nanocrystals as Heterogeneous Catalysts. Heliyon 2023, 9 (10), e21038 10.1016/j.heliyon.2023.e21038. 37920271 PMC10618990

[ref21] Aljammal N.; Lauwaert J.; Biesemans B.; Vandevyvere T.; Sabbe M. K.; Heynderickx P. M.; Thybaut J. W. UiO-66 Metal-Organic Frameworks as Aldol Condensation Catalyst: Impact of Defects, Solvent, Functionality on the Catalytic Activity and Selectivity. J. Catal. 2024, 433, 115471 10.1016/j.jcat.2024.115471.

[ref22] Liu X.; Wang H.; Liu X.; Yang F.; Guan L.; Sani S.; Sun C.; Wu Y. Development of MgSO4/Mesoporous Silica Composites for Thermochemical Energy Storage: The Role of Porous Structure on Water Adsorption. Energy Rep. 2022, 8, 4913–4921. 10.1016/j.egyr.2022.03.137.

[ref23] Shkatulov A.; Joosten R.; Fischer H.; Huinink H. Core-Shell Encapsulation of Salt Hydrates into Mesoporous Silica Shells for Thermochemical Energy Storage. ACS Appl. Energy Mater. 2020, 3 (7), 6860–6869. 10.1021/acsaem.0c00971.

[ref24] Dindar M. H.; Yaftian M. R.; Rostamnia S. Potential of Functionalized SBA-15 Mesoporous Materials for Decontamination of Water Solutions from Cr(VI), As(V) and Hg(II) Ions. J. Environ. Chem. Eng. 2015, 3 (2), 986–995. 10.1016/j.jece.2015.03.006.

[ref25] Dobrzyńska J.; Olchowski R.; Zięba E.; Dobrowolski R. A Hybrid Zr/Amine-Modified Mesoporous Silica for Adsorption and Preconcentration of as before Its FI HG AAS Determination in Water. Microporous Mesoporous Mater. 2021, 328, 111484 10.1016/j.micromeso.2021.111484.

[ref26] Martín A.; Arsuaga J. M.; Roldán N.; Martínez A.; Sotto A. Effect of Amine Functionalization of SBA-15 Used as Filler on the Morphology and Permeation Properties of Polyethersulfone-Doped Ultrafiltration Membranes. J. Membr. Sci. 2016, 520, 8–18. 10.1016/j.memsci.2016.07.040.

[ref27] Morales V.; Martín A.; Ortiz-Bustos J.; Sanz R.; García-Muñoz R. A. Effect of the Dual Incorporation of Fullerene and Polyethyleneimine Moieties into SBA-15 Materials as Platforms for Drug Delivery. J. Mater. Sci. 2019, 54 (17), 11635–11653. 10.1007/s10853-019-03708-0.

[ref28] García-Fernández A.; Sancenón F.; Martínez-Máñez R. Mesoporous Silica Nanoparticles for Pulmonary Drug Delivery. Adv. Drug Delivery Rev. 2021, 177, 113953 10.1016/j.addr.2021.113953. 34474094

[ref29] Van Grieken R.; Martínez F.; Morales G.; Martín A. Nafion-Modified Large-Pore Silicas for the Catalytic Acylation of Anisole with Acetic Anhydride. Ind. Eng. Chem. Res. 2013, 52 (30), 10145–10151. 10.1021/ie401360b.

[ref30] Hoffmann F.; Cornelius M.; Morell J.; Fröba M. Silica-Based Mesoporous Organic-Inorganic Hybrid Materials. Angew. Chem., Int. Ed. 2006, 45 (20), 3216–3251. 10.1002/anie.200503075. 16676373

[ref31] Nelson Appaturi J.; Andas J.; Ma Y.-K.; Lee Phoon B.; Muazu Batagarawa S.; Khoerunnisa F.; Hazwan Hussin M.; Ng E.-P. Recent Advances in Heterogeneous Catalysts for the Synthesis of Alkyl Levulinate Biofuel Additives from Renewable Levulinic Acid: A Comprehensive Review. Fuel 2022, 323, 124362 10.1016/j.fuel.2022.124362.

[ref32] Appaturi J. N.; Johan M. R.; Ramalingam R. J.; Al-Lohedan H. A.; Vijaya J. J. Efficient Synthesis of Butyl Levulinate from Furfuryl Alcohol over Ordered Mesoporous Ti-KIT-6 Catalysts for Green Chemistry Applications. RSC Adv. 2017, 7 (87), 55206–55214. 10.1039/C7RA10289E.

[ref33] Ristiana D. D.; Suyanta S.; Nuryono N. Sulfonic Acid-Functionalized Silica with Controlled Hydrophobicity as an Effective Catalyst for Esterification of Levulinic Acid. Mater. Today Commun. 2022, 32, 103953 10.1016/j.mtcomm.2022.103953.

[ref34] Zanuttini M. S.; Tonutti L. G.; Neyertz C. A.; Ferretti C.; Sánchez B.; Dalla Costa B. O.; Querini C. A. Production of a High Molecular Weight Jet-Fuel Precursor from Biomass Derived Furfural and 2-Methylfuran Using Propyl Sulfonic SBA-15 Catalysts. Appl. Catal., A 2023, 665, 119383 10.1016/j.apcata.2023.119383.

[ref35] Leo P.; Crespí N.; Palomino C.; Martín A.; Orcajo G.; Calleja G.; Martinez F. Catalytic Activity and Stability of Sulfonic-Functionalized UiO-66 and MIL-101 Materials in Friedel-Crafts Acylation Reaction. Catal. Today 2022, 390–391, 258–264. 10.1016/j.cattod.2021.10.007.

[ref36] Wawrzyńczak A.; Jarmolińska S.; Nowak I. Nanostructured KIT-6 Materials Functionalized with Sulfonic Groups for Catalytic Purposes. Catal. Today 2022, 397, 526–539. 10.1016/j.cattod.2021.06.019.

[ref37] Paniagua M.; Cuevas F.; Morales G.; Melero J. A. Sulfonic Mesostructured SBA-15 Silicas for the Solvent-Free Production of Bio-Jet Fuel Precursors via Aldol Dimerization of Levulinic Acid. ACS Sustain. Chem. Eng. 2021, 9 (17), 5952–5962. 10.1021/acssuschemeng.1c00378.

[ref38] Margolese D.; Melero J. A.; Christiansen S. C.; Chmelka B. F.; Stucky G. D. Direct Syntheses of Ordered SBA-15 Mesoporous Silica Containing Sulfonic Acid Groups. Chem. Mater. 2000, 12 (8), 2448–2459. 10.1021/cm0010304.

[ref39] Melero J. A.; Stucky G. D.; van Grieken R.; Morales G. Direct Syntheses of Ordered SBA-15 Mesoporous Materials Containing Arenesulfonic Acid Groups. J. Mater. Chem. 2002, 12 (6), 1664–1670. 10.1039/b110598c.

[ref40] Martínez F.; Morales G.; Martín A.; van Grieken R. Perfluorinated Nafion-Modified SBA-15 Materials for Catalytic Acylation of Anisole. Appl. Catal., A 2008, 347 (2), 169–178. 10.1016/j.apcata.2008.06.015.

[ref41] Huo Q.; Margolese D. I.; Stucky G. D. Surfactant Control of Phases in the Synthesis of Mesoporous Silica-Based Materials. Chem. Mater. 1996, 8 (5), 1147–1160. 10.1021/cm960137h.

[ref42] Zhao D.; Feng J.; Huo Q.; Melosh N.; Fredrickson G. H.; Chmelka B. F.; Stucky G. D. Triblock Copolymer Syntheses of Mesoporous Silica with Periodic 50 to 300 Angstrom Pores. Science 1998, 279 (5350), 548–552. 10.1126/science.279.5350.548. 9438845

[ref43] Martín A.; Morales G.; Martínez F.; Van Grieken R.; Cao L.; Kruk M. Acid Hybrid Catalysts from Poly(Styrenesulfonic Acid) Grafted onto Ultra-Large-Pore SBA-15 Silica Using Atom Transfer Radical Polymerization. J. Mater. Chem. 2010, 20 (37), 8026–8035. 10.1039/c0jm01589j.

[ref44] Kleitz F.; Solovyov L. A.; Anilkumar G. M.; Choi S. H.; Ryoo R. Transformation of Highly Ordered Large Pore Silica Mesophases (Fm3m, Im3m and P6mm) in a Ternary Triblock Copolymer-Butanol-Water System. Chem. Commun. 2004, (13), 1536–1537. 10.1039/B403903C. 15216368

[ref45] Cao L.; Kruk M. Short Synthesis of Ordered Silicas with Very Large Mesopores. RSC Adv. 2014, 4 (1), 331–339. 10.1039/C3RA44203A.

[ref46] Wang R.; Shen F.; Tang Y.; Guo H.; Lee Smith R.; Qi X. Selective Conversion of Furfuryl Alcohol to Levulinic Acid by SO3H-Containing Silica Nanoflower in GVL/H2O System. Renewable Energy 2021, 171, 124–132. 10.1016/j.renene.2021.02.064.

[ref47] Cao L.; Kruk M. Synthesis of Large-Pore SBA-15 Silica from Tetramethyl Orthosilicate Using Triisopropylbenzene as Micelle Expander. Colloids Surf., A 2010, 357 (1–3), 91–96. 10.1016/j.colsurfa.2009.09.019.

[ref48] Kleitz F.; Kim T.-W.; Ryoo R. Phase Domain of the Cubic *Im*3̅*m* Mesoporous Silica in the EO_106_PO_70_EO_106_–Butanol–H_2_O System3̅m Mesoporous Silica in the EO106PO70EO106-Butanol-H2O System. Langmuir 2006, 22 (1), 440–445. 10.1021/la052047+. 16378457

[ref49] Kruk M.; Hui C. M. Synthesis and Characterization of Large-Pore FDU-12 Silica. Microporous Mesoporous Mater. 2008, 114 (1–3), 64–73. 10.1016/j.micromeso.2007.12.015.

[ref50] Göltner C. G.; Smarsly B.; Berton B.; Antonietti M. On the Microporous Nature of Mesoporous Molecular Sieves. Chem. Mater. 2001, 13 (5), 1617–1624. 10.1021/cm0010755.

[ref51] Chen F.; Xu X.-J.; Shen S.; Kawi S.; Hidajat K. Microporosity of SBA-3 Mesoporous Molecular Sieves. Microporous Mesoporous Mater. 2004, 75 (3), 231–235. 10.1016/j.micromeso.2004.07.028.

[ref52] van Grieken R.; Calleja G.; Stucky G. D.; Melero J. A.; García R. A.; Iglesias J. Supercritical Fluid Extraction of a Nonionic Surfactant Template from SBA-15 Materials and Consequences on the Porous Structure. Langmuir 2003, 19 (9), 3966–3973. 10.1021/la026970c.

[ref53] Lorenzo D.; Simón E.; Santos A.; Romero A. Kinetic Model of Catalytic Self-Condensation of Cyclohexanone over Amberlyst 15. Ind. Eng. Chem. Res. 2014, 53 (49), 19117–19127. 10.1021/ie5032265.

[ref54] Mahajan Y. S.; Kamath R. S.; Kumbhar P. S.; Mahajani S. M. Self-Condensation of Cyclohexanone over Ion Exchange Resin Catalysts: Kinetics and Selectivity Aspects. Ind. Eng. Chem. Res. 2008, 47 (1), 25–33. 10.1021/ie061275b.

[ref55] Li G.; Wang B.; Kobayashi T.; Pruski M.; Resasco D. E. Optimizing the Surface Distribution of Acid Sites for Cooperative Catalysis in Condensation Reactions Promoted by Water. Chem Catal. 2021, 1 (5), 1065–1087. 10.1016/j.checat.2021.08.005.

[ref56] Li G.; Wang B.; Chen B.; Resasco D. E. Role of Water in Cyclopentanone Self-Condensation Reaction Catalyzed by MCM-41 Functionalized with Sulfonic Acid Groups. J. Catal. 2019, 377, 245–254. 10.1016/j.jcat.2019.07.032.

[ref57] Morales G.; Athens G.; Chmelka B.; Van Grieken R.; Melero J. Aqueous-Sensitive Reaction Sites in Sulfonic Acid-Functionalized Mesoporous Silicas. J. Catal. 2008, 254 (2), 205–217. 10.1016/j.jcat.2007.12.011.

