# Supplementary material for: Efficient Self-Condensation of Cyclohexanone into Biojet Fuel Precursors over Sulfonic Acid-Modified Silicas: Insights on the Effect of Pore Size and Structure
Source: ACS Sustain Chem Eng. 2024 Jun 24;12(27):10175–85. doi: 10.1021/acssuschemeng.4c01956 (PMC11234364; doi:10.1021/acssuschemeng.4c01956)
Supplement: Supplementary file 1 — sc4c01956_si_001.pdf [file sc4c01956_si_001.pdf]

Supporting Information

Efficient self-condensation of cyclohexanone into bio-jet fuel precursors over sulfonic acid-modified silicas: insights on the effect of pore size and structure

Antonio Martín,[a,*] Esther Arribas-Yuste,[a] Marta Paniagua,[a] Gabriel Morales,[a,b] Juan A. Melero[a,b]

[a] Chemical and Environmental Engineering Group. ESCET, Universidad Rey Juan Carlos. c/Tulipán s/n 28933 Móstoles, Spain.

[b] Instituto de Tecnologías para la Sostenibilidad (ITPS). ESCET, Universidad Rey Juan Carlos. c/Tulipán s/n 28933 Móstoles, Spain.

KEYWORDS: Mesoporous silica; sulfonic acid catalysts; aldol condensation; cyclohexanone; bio-jet fuel.

* Corresponding author:

E-mail: antonio.martin.rengel@urjc.es

Phone-number: +34-91 488 70 85

Supporting information contents:

Number of pages: 3

Number of figures: 2

Number of tables: 1

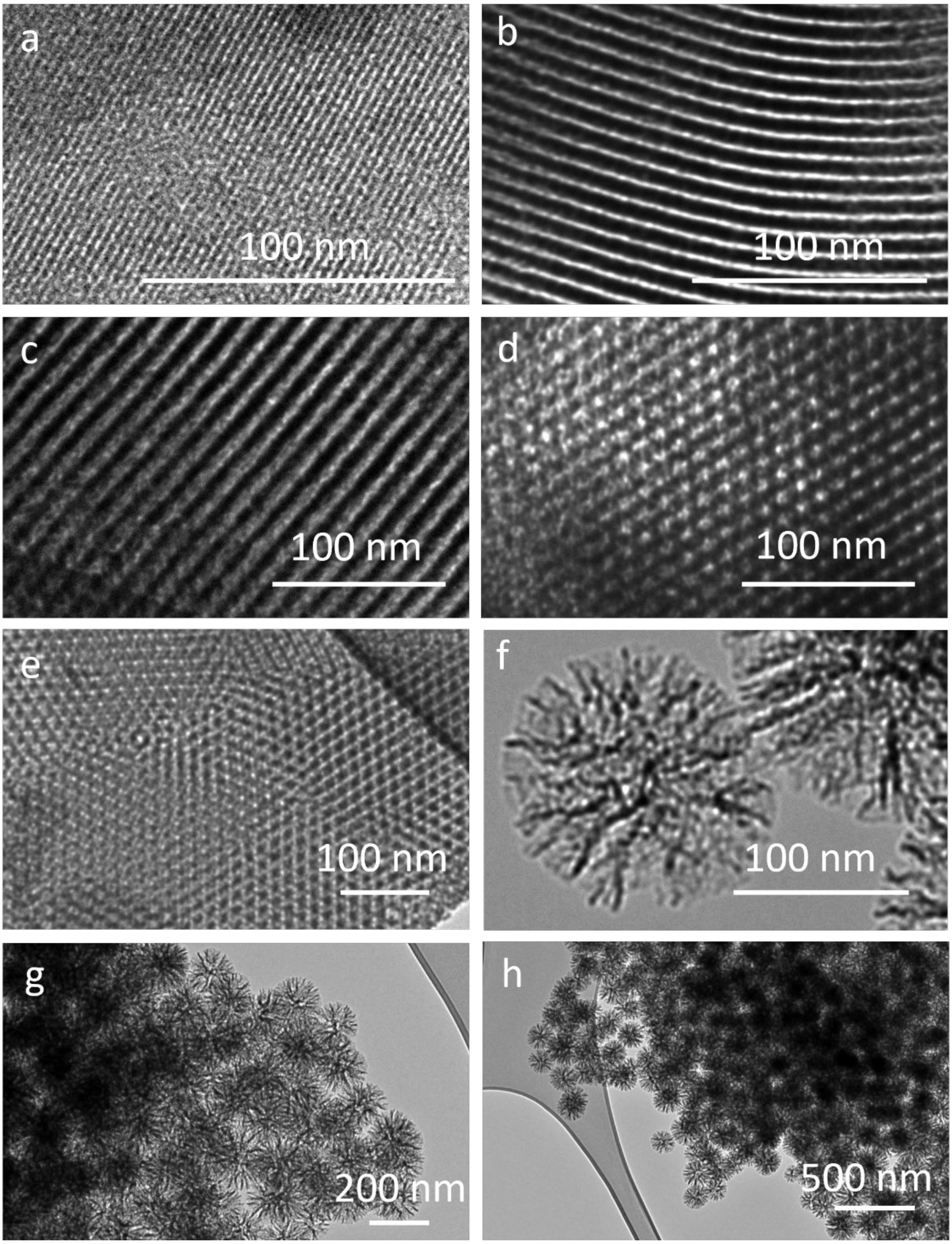

Figure S1. TEM images of the synthetized silica supports: SBA-3 (a), SBA-15 (b), LP-SBA-15 (c), SBA-16 (d), FDU-12 (e) and SiNF (f-g-h).

Table S1. Effect of the temperature on the catalytic performance using Amberlyst-15 as catalyst (catalyst loading: 1 wt.% based on CHO mass, solventless conditions, reaction time: 5h).

**Temperature (ºC)**	Conversion of cyclohexanone (%)	Selectivity towards the two dimers (%)
80	33.7	96.1
100	44.2	95.2
120	50.1	82.5

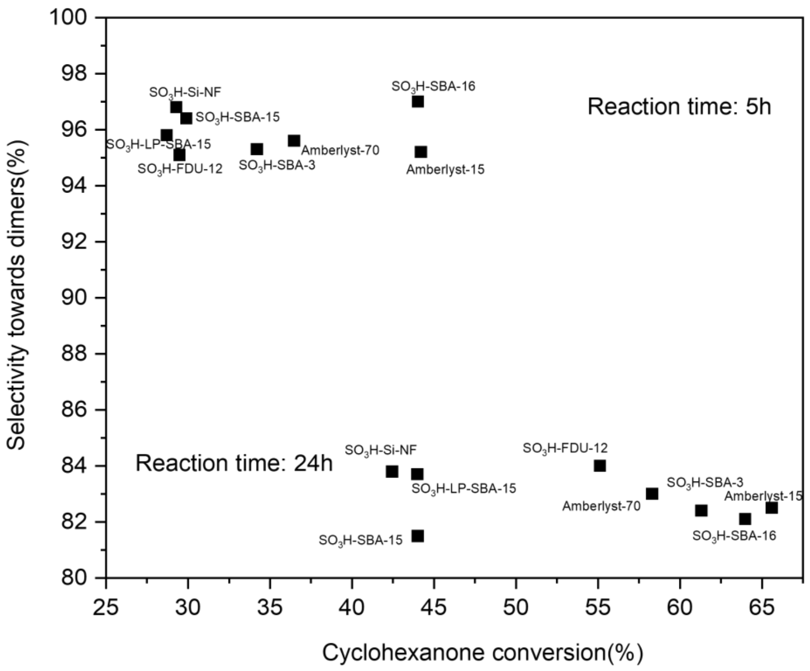

Figure S2. Conversion of cyclohexanone versus selectivity towards dimers at 5 h and 24 h. Temperature: 100 °C, catalyst loading: 1 wt.% based on CHO mass, solventless conditions.
